# Computational identification of vesicular transport proteins from sequences using deep gated recurrent units architecture

**DOI:** 10.1016/j.csbj.2019.09.005

**Published:** 2019-10-25

**Authors:** Nguyen Quoc Khanh Le, Edward Kien Yee Yapp, N. Nagasundaram, Matthew Chin Heng Chua, Hui-Yuan Yeh

**Affiliations:** a Medical Humanities Research Cluster, School of Humanities, Nanyang Technological University, 48 Nanyang Ave, 639818, Singapore; b Professional Master Program in Artificial Intelligence in Medicine, Taipei Medical University, Taipei 106, Taiwan; c Singapore Institute of Manufacturing Technology, 2 Fusionopolis Way, #08-04, Innovis, 138634, Singapore; d Institute of Systems Science, 25 Heng Mui Keng Terrace, National University of Singapore, 119615, Singapore

**Keywords:** Vesicular trafficking model, Protein function prediction, Transport proteins, Recurrent neural network, Deep learning, Membrane proteins

## Abstract

Protein function prediction is one of the most well-studied topics, attracting attention from countless researchers in the field of computational biology. Implementing deep neural networks that help improve the prediction of protein function, however, is still a major challenge. In this research, we suggested a new strategy that includes gated recurrent units and position-specific scoring matrix profiles to predict vesicular transportation proteins, a biological function of great importance. Although it is difficult to discover its function, our model is able to achieve accuracies of 82.3% and 85.8% in the cross-validation and independent dataset, respectively. We also solve the problem of imbalance in the dataset via tuning class weight in the deep learning model. The results generated showed sensitivity, specificity, MCC, and AUC to have values of 79.2%, 82.9%, 0.52, and 0.861, respectively. Our strategy shows superiority in results on the same dataset against all other state-of-the-art algorithms. In our suggested research, we have suggested a technique for the discovery of more proteins, particularly proteins connected with vesicular transport. In addition, our accomplishment could encourage the use of gated recurrent units architecture in protein function prediction.

## Graphical abstract

**Figure:**
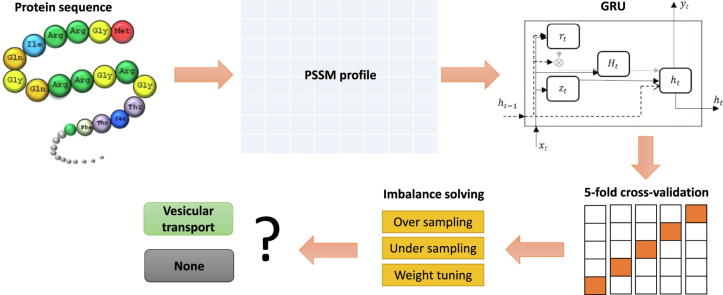

## 1 Introduction

Proteins perform a wide variety of functions within different eukaryotic cell compartments. Therefore, prediction of protein functions is the most well-studied problems in computational biology field, attracting the attention of countless scientists. With a multitude of computational methods, much attention has been provided to enhance the predictive efficiency of protein functions. To tackle this problem, there are two popular solutions: finding the finest attribute sets and producing powerful predictive neural networks. For example, in the past, some bioinformatics researchers used machine learning techniques with a strong feature set such as pseudo amino acid composition [1], [2], position-specific scoring matrix (PSSM) [3], [4], and biochemical properties [5], [6]. Nowadays, with the rise of deep learning, many researchers in the field of biology have been attempting to apply it to the prediction of protein functions. There has been much research done on the application of deep neural networks in predicting different functions of proteins, such as electron transport chain [7], human protein subcellular localization [8] and Rab GTPases [9]. However, it requires a lot of efforts to create innovative deep neural networks and to enhance the performance results. In this study, we propose a novel approach to address this issue by using deep gated recurrent unit (GRU) structure, which is a form of deep neural network. GRU has been applied in a variety of fields, achieving high performing results. Thus, we now extend it into computational biology via high throughput sequencing data. To explain in detail, we applied our techniques in predicting the vesicular transport protein, which is one of the most important molecules in transmembrane.

A vesicular transport protein, or the so-called vesicular transporter, is a protein, contained in the cell membrane, which organizes or promotes the activities of explicit molecules across a vesicle's membrane. It plays a vital function in the intracellular transport of molecules crosswise over membranes. Accordingly, vesicular transporters oversee the centralization of molecules inside a vesicle. Vesicular transport is thus a primary cellular compartment, in charge of trafficking molecules between different explicit membrane-enclosed components. The selectivity of such transport is, therefore, key to maintaining the functional organization of the cell.

Abnormal vesicular transport proteins have been shown to be associated with a lot of human diseases. In [10], authors reviewed the mechanism of vesicular transport proteins and their role in synaptic transmission, behavior, and neural degeneration. Vesicular transport protein mutations occur in many genetic disorders and provide insights into the molecular pathology of popular multifactorial diseases associated with disordered trafficking mechanisms [11]. In [12], many human diseases caused by abnormal vesicular transport protein were reported, e.g., Hermansky–Pudlak syndrome, Cranio‐lenticulo‐sutural dysplasia, Chylomicron retention disease, and so on. It also participated in disease pathogenesis of Alzheimer’s disease [13]. Vesicular transport protein structure has also been used to design the Glatiramer drug, which is also used in the treatment of patients with recurrent multiple sclerosis [14].

Due to the significant role that the vesicular transporter plays in the functioning and structuring of eukaryotic cells, much progress toward elucidating the molecular mechanisms of vesicular transport proteins has been made in the area of cell biology research such as emerging inductive technology, mass spectrometry-based proteomics [15], [16], Morpholino knockdown [17], dissection [18], and gene expression [19]. The use of these experimental techniques, however, is costly and time-consuming. Therefore, in investigating and characterizing vesicular transport proteins, there is a need to find new computational approaches to supplant the experimental techniques. Furthermore, since more protein sequences have been found with the development of protein sequencing techniques, the amount of protein sequence entries is now a thousand times higher than the amount of entries from around 25 years ago. Faced with the rise of new protein sequences found in the post-genomic age, there was a desire to develop automated computational prediction methods to identify vesicular transport proteins quickly and accurately.

There are few computational studies to investigate the biological processes or molecular functions that relates to vesicular transport proteins. For example, one of the most common research is TCDB [20], a web-accessible, curated, relational database comprising of sequence, classification, structural, functional and evolutionary transport system data, including vesicular transport proteins from a multitude of living organisms. Going in-depth regarding the discussion of vesicular transport proteins, there have been a few researchers that attempted to identify some of the proteins. Anderson and Sandelius [21], for example, searched for the chloroplast-localized homologues of cytosolic vesicular trafficking components in the Arabidopsis thaliana genome by using web-based subcellular prediction tools. Emelie et al. [22] used bioinformatics analysis to indicate the role of two common vesicular transport proteins (Coat and Clathrin). Another kind of vesicular transport proteins is SNARE, which has been investigated in [23], [24], [25]. In order to classify the molecular function of Rab GTPases in vesicular transport system, Le et al. [9] created a computational model by using 2D convolutional neural network (CNN) and PSSM profiles. However, all current published works only focus on the whole transport protein system or on one specific type of vesicular transport protein. Therefore, identifying vesicular transport proteins has not yet been attained and the present study attempts to deal with this problem.

By using multiple representations of features and neural networks, there have recently been a lot of research addressing the issue of protein function prediction. PSSM profile is one of the most popular characteristics that can solve the issue while delivering high performance. Most of these studies undertaken, however, did not fully exploit the benefits of PSSM profiles in deep neural networks. In the previous works, the PSSM profiles had been scaled to a fixed length to feed into the neural networks and then performed classification. But the ordering information was missed in the process and it affects the outcomes of the performance. To address this issue, the incorporation of 1D CNN and GRU has been applied in this study. GRU architecture has indeed been used in computational biology problems such as protein sequence [26], [27], [28] and RNA sequence [29]. To our understanding, no prior computational study has specifically integrated the GRU and PSSM profiles in the vesicular transport protein prediction. Some essential contributions of this study to its field are as follows: (1) an innovative computational model for the identification of vesicular transportation proteins showing powerful improvements beyond the previous models; (2) a benchmark dataset and new discovered data for further study on vesicular transport protein, and (3) a study that would provide biologists and researchers with a great deal of information as they better understand the vesicular transportation protein structures and conduct future research.

## 2 Materials and methods

**Fig. 1:**
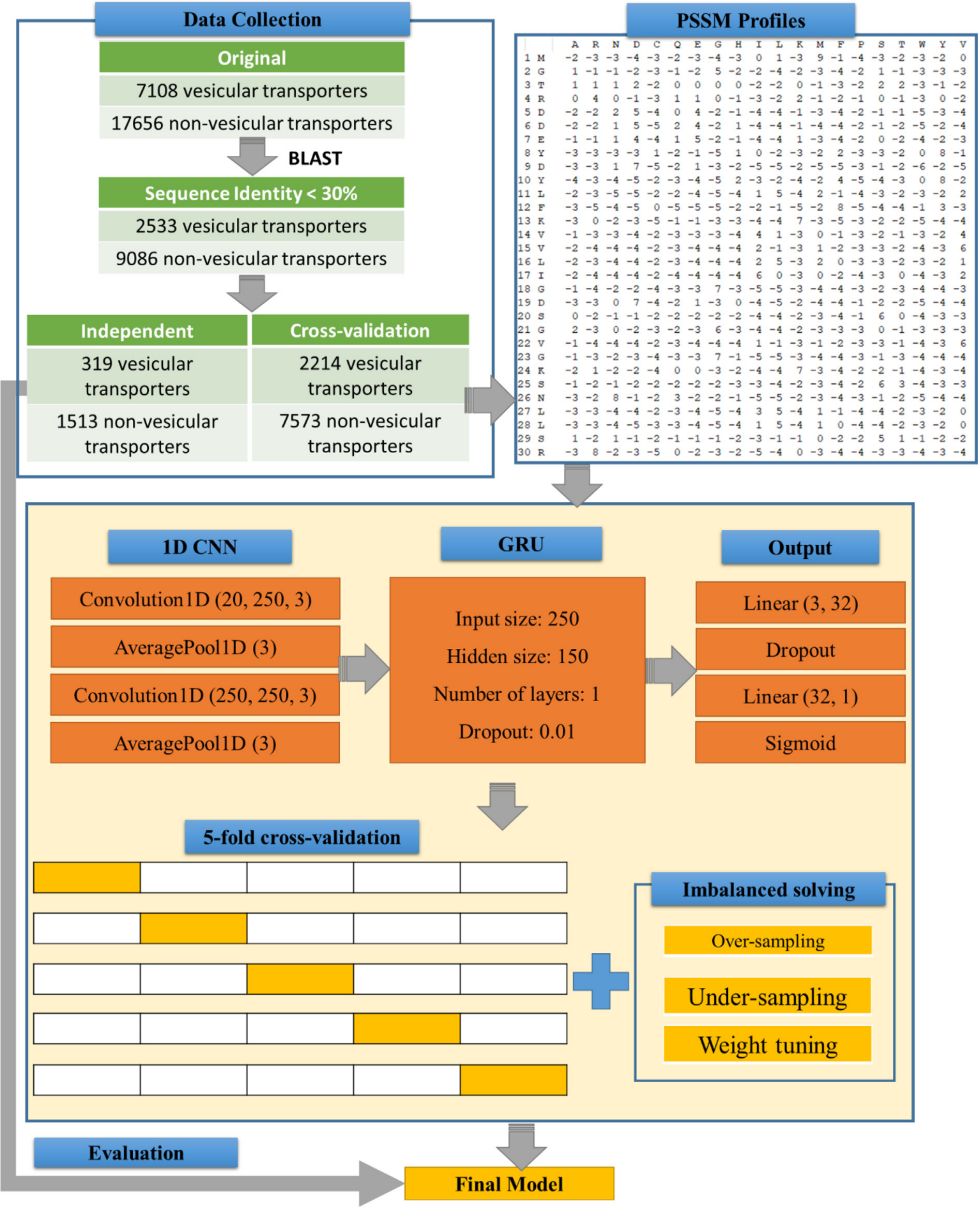
The flowchart for identifying vesicular transport proteins using GRU and PSSM profiles.

Our flowchart is illustrated in Fig. 1 and the details of it was described in the following sub-sections as follows.

### 2.1 Benchmark dataset

For an accurate and fair classification problem, data collection plays a very important role. In this study, we collected data from UniProt [30] (release 2018_07) and Gene Ontology (GO) [31], which provides high-quality resources for research on gene products. We performed the following steps:

(1) We collected protein sequences through searching the UniProt database [30] (release 2018_07) with keyword “vesicular transport” or gene ontology terms “vesicular transport”, and then, the initial positive dataset for vesicular transport protein was created.
(2) Note that we only chose the reviewed proteins which have been annotated by biological experiments. It means that we filtered out all non-experimentally validated terms from both UniProt and GeneOntology.
(3) From the datasets, we eliminated the homologous sequences to ensure that any two sequences shared a pairwise sequence identity of less than 30%. To perform this task, we used BLAST [32] with a cut-off level of 30%.
(4) In the last step, we removed all the protein sequences with non-canonical amino acids (e.g. X, U, B, and Z).

Our proposed study is regarding a binary classification problem between vesicular transport proteins and non-vesicular transport proteins, so that a set of general proteins were collected as negative data. In this work, we chose the membrane protein, which is a big family of general protein containing a lot of protein functions (including vesicular transport). Briefly, we extracted all of the membrane proteins in UniProt and excluded the vesicular transport proteins. Similar to the previous step, only reviewed proteins and canonical amino acids were retained and we also used the BLAST clustering [32] with a cut-off level of 30% to remove the highly similar sequences.

In summary, we received 2533 vesicular transport proteins and 9086 non-vesicular transport proteins in all species. To conduct the experiments, the data was divided into two sets: cross-validation and independent data set. The cross-validation dataset was used for the construction of our model, and the independent dataset was used for evaluating the performance of the proposed method. To separate these two sets, we randomly picked the newly discovered proteins (by 2009) as independent dataset, and the rest of sequences was used as cross-validation dataset. Since we used the year of 2009 as a cut-off point, therefore, there was a different class distribution in cross-validation and independent datasets. Table 1 lists all the details of the dataset using in this study. We also provided our benchmark dataset for further study at https://github.com/khanhlee/vesicular-gru/tree/master/data.

**Table 1:** Statistics of all dataset used in this study.

	Original	Identity < 30%	Cross-validation	Independent
Vesicular transport	7108	2533	2214	319
Non-vesicular transport	17656	9086	7573	1513

### 2.2 Construction of PSSM profiles

In this study, we extracted features using PSSM profile, which is a well-known representation of patterns in protein sequences. As mentioned in the original paper [3], it is used to decode the evolutionary information of proteins. A PSSM for a protein is an N*20 matrix, in which N is the sequence length of the query protein. It assigns a P_ij_ score for the j^th^ amino acid in the i^th^ position of the query sequence with a high value that indicates a highly conserved position and a low value that indicates a weakly conservative. Since its discovery, it has been used in numerous studies in bioinformatics with valuable results [33], [34], [35]. This study used PSI-BLAST (in BLAST package [32]) to search all sequences one-by-one against non-redundant (NR) database with two iterations and e-value threshold of 0.001. Thereafter, the PSSM profiles have been generated and used for the next experiments.

### 2.3 Deep gated recurrent units architecture

After generating PSSM profiles from FASTA sequences, we used them as features to be inserted into our deep neural networks. To extract the features in PSSM profiles, we applied GRU architecture, which is a type of recurrent neural network (RNN) that has been used in various bioinformatics applications such as predicting protein secondary structure [36], classifying widely and rarely expressed genes [37], biomedical named entity recognition [38]. The big advantage of this architecture is that it can work well with sequential data and accept an input with different lengths. Therefore, it can be suitable with our kind of data. These networks are at the heart of speech recognition, translation and more.

We used PyTorch [39] as our deep learning framework for implementing our GRU structure. NVIDIA Titan XP was used to accelerate the graphic processing unit (GPU) via CUDA platform. In the first initiation, we extracted the information from PSSM profiles by using a 1D CNN over an input shape. Given an input size ((*N,C_in_,L*), we are able to exactly calculate the output (*N,C_out_,L_out_*) by using the following formula:

(1) $$out\left(N_{i},C_{{\mathrm{out}}_{j}}\right)=bias\left(C_{{\mathrm{out}}_{j}}\right)+\sum_{k=0}^{C_{\mathrm{in}}-1}weight\left(C_{{\mathrm{out}}_{j}},k\right)*input\left(N_{i},k\right)$$

where *N* is a batch size, *C* is the channel number, *L* is a length of the signal sequence, and * is the valid cross-correlation operator. In this architecture, we limited the input size to be equalled with the number of amino acids (=20). For this step, we put an input shape (*N, C_in_, L_in_*) to give an output shape (*N, C_out_, L_out_*) where:

(2) $$L_{\mathrm{out}}=\left\lfloor \frac{L_{\mathrm{in}}+2*padding-dilation*\left(kernel_{\mathrm{size}}-1\right)-1}{\mathrm{stride}}+1\right\rfloor$$

An important benefit of inputting all the PSSM profiles into the neural network is that it prevents missing information of PSSM profiles. Next, the pooling layer takes a sliding window or a certain region through the input matrix, which transforms the values into representative values. The transformation is carried out either by taking the maximum value (max pooling) or the average of the values (average pooling) in the window. In our study, we performed a 1D average pooling over an input of several values. In this step, we can also calculate the output (*N, C, L*) and kernel size *k* as follows:

(3) $$out\left(N_{i},C_{j},l\right)=\frac{1}{k}\sum_{m=0}^{k}input\left(N_{i},C_{j},stride*l+m\right)$$

Zero-padding is the method of symmetrically adding zeros to the input matrix, making it possible to adjust the size of the input to certain demands. Zero values were added at the start and end of the matrices in the model described in the present research. This enabled us to apply the filter to the matrix boundary positions. If the padding size is not zero, the input is implicitly zero-padded to padd on both sides the amount of points. It is possible to calculate the input shape (*N, C, L_in_*) and output shape (*N, C, L_out_*) by:

(4) $$L_{\mathrm{out}}=\left\lfloor \frac{L_{\mathrm{in}}+2+padding-kernel\_size}{\mathrm{stride}}+1\right\rfloor$$

A multi-layer GRU was implemented after the generation of feature sets with 1D CNN. GRU is an enhanced version of the recurrent neural network. GRU utilizes the so-called update gate and reset gate to fix the disappearing gradient issue of a conventional RNN. The concept behind a GRU layer, as well as their resulting equations, is quite comparable to that of an LSTM layer. As described in the previous works [27], [28], each layer of GRU cells was calculated according to the following functions:

(1) Update gate helps the model determine how much of the past information (from previous steps in time) needs to be passed on to the future. We used the formula to calculate the update door *z_t_* for time step *t*:

(5) $$z_{t}=\sigma \left(W_{\mathrm{iz}}x_{t}+b_{\mathrm{iz}}+W_{\mathrm{hz}}h_{\left(t-1\right)}+b_{\mathrm{hz}}\right)$$

where *x_t_* is the input at time *t*, *h_(t−1)_* is the hidden state of the previous layer at time *t-1* or the initial hidden state at time *0*, *σ* is the sigmoid function, *W* is weight, and *b* is bias

(2) Reset gate is used from the model to determine how much of the prior data should be forgotten. We use the following formula to calculate it:

(6) $$r_{t}=\sigma \left(W_{\mathrm{ir}}x_{t}+b_{\mathrm{ir}}+W_{\mathrm{hr}}h_{\left(t-1\right)}+b_{\mathrm{hr}}\right)$$

(3) Current memory content stores appropriate data from the past using the reset gate.

(7) $$n_{t}=\tanh\left(W_{\mathrm{in}}x_{t}+b_{\mathrm{in}}+r_{t}\left(W_{\mathrm{hn}}h_{\left(t-1\right)}+b_{\mathrm{hn}}\right)\right)$$

(4) Final memory at the present time step: as the last phase, the network needs to calculate the *h_t_* vector that retains the present unit's data and transfers it to the network. The update gate is required to do this. The following is performed:

(8) $$h_{t}=\left(1-z_{t}\right)n_{t}+z_{t}h_{\left(t-1\right)}$$

### 2.4 Output layers

In the output layers, we firstly applied non-linear activation layer, namely sigmoid. Commonly, it is problematic in RNN and it applies the element-wise function as follows:

(9) $$Sigmoid\left(x\right)=\frac{1}{1+exp(-x)}$$

Then we used linear layers to apply a linear transformation to the incoming data:

(10) y=Ax+b

In summary, the output shape of linear layers can be described as:

(1) Input: ((N,∗,in_features) where ∗ indicates any additional dimensions number.
(2) Output: (N,∗,out_features) where all dimensions have the same shape as the input except the last dimension.

We next applied a dropout layer for regularization and prevention of neuron co-adaptions [40]. This layer also plays an important role in helping our model prevent overfitting. The dropout values in this study range from 0 to 1 to evaluate our model. Given *p* as the dropout values, we can calculate the output of this layer via scaled function:

(11) $$\mathrm{out}=\frac{1}{1-p}$$

Finally, Table 2 summarizes all sections of our GRU model with weights and trainable parameters (434,365 parameters).

**Table 2:** Summary of GRU architecture in this study.

Layer	Weights	Parameters
Conv1d (20, 250, 3)	((250, 20, 3), (250,))	15,250
AvgPool1d (3)	0	0
Conv1d (250, 250, 3)	((250, 250, 3), (250,))	187,750
AvgPool1d (3)	0	0
GRU (250, 150, 1)	((750, 150), (750, 150), (750,), (750,))	226,500
Linear (150, 32)	((32, 150), (32,))	4832
Dropout (0.01)	0	0
Linear (32, 1)	((1, 32), (1,))	33
Sigmoid ()	0	0

### 2.5 Assessment of predictive ability

The main aim of this research is to predict whether an unknown sequence is a vesicular transport protein; therefore, we used “positive” to describe the vesicular transport protein, and “negative” to describe the non-vesicular transport protein. Although the jackknife test is an approximately unbiased performance generalization estimator, it has two major drawbacks, e.g. it has high variance (because all the data sets used for the estimation are very similar) and it is also expensive to calculate (it requires n estimates, where n is the number of observations in the dataset) [41]. Therefore, it has been proposed that 5 or 10 fold cross-validation is a good compromise between unbiasedness and computational requirements. Moreover, there are resources to learn more about it [42]. We thus trained our model by using 5-fold cross-validation method for the entire training dataset. We have performed 10 times of 5-fold cross-validation to obtain more accurate outcomes, since 5-fold cross-validation results differently each time. The final result of cross-validation is then the average result of all the 10 times of 5-fold cross-validation testings. Hyperparameter optimization method was used to discover the best model for each dataset based on the 5-fold cross-validation tests. In addition, the independent data set was utilized to assess the results precision in order to regulate any systematic bias in the cross-validation set. In this examination, the default threshold of 0.5 was selected for binary classification.

For evaluating the performance of the methods, we adopted Chou’s criterion used in many computational biology studies [41], [43]. Since Chou introduced this set of intuitive metrics, they have been concurred and admired by a series of recent publications because of their improvement from the traditional metrics. They provided the intuitiveness and were easily comprehensible for all biologists. These intuitive metrics include sensitivity, specificity, accuracy, and Matthews correlation coefficient (MCC) were calculated by the following formulas: (TP, FP, TN, FN are true positive, false positive, true negative, and false negative values, respectively):

(12) $$\mathrm{Sensitivity}=1-\frac{N_{-}^{+}}{N^{+}},\;0\leq Sen\leq 1$$

(13) $$\mathrm{Specificity}=1-\frac{N_{+}^{-}}{N^{-}},\;0\leq Spec\leq 1$$

(14) $$\mathrm{Accuracy}=1-\frac{N_{-}^{+}+N_{+}^{-}}{N^{+}+N^{-}},\;0\leq Acc\leq 1$$

(15) $$\mathrm{MCC}=\frac{1-\left(\frac{N_{-}^{+}}{N^{+}}+\frac{N_{+}^{-}}{N^{-}}\right)}{\sqrt{\left(1+\frac{N_{+}^{-}-N_{-}^{+}}{N^{+}}\right)\left(1+\frac{N_{-}^{+}-N_{+}^{-}}{N^{-}}\right)}},\;-1\leq MCC\leq 1$$

where:

(16) $$\left\{\begin{array}{c} N_{+}^{-}=FP\\ N_{-}^{+}=FN\\ \begin{array}{c} N^{+}=TP+N_{-}^{+}\\ N^{-}=TN+N_{+}^{-} \end{array} \end{array}\right)$$

Furthermore, since our problem is a class-imbalanced problem, we also analyzed the Precision metric as follows:

(17) $$\mathrm{Precision}=\frac{\mathrm{TP}}{\mathrm{TP}+FP}$$

## 3 Results and discussions

### 3.1 Comparison between vesicular transport proteins and non-vesicular transport proteins

**Fig. 2:**
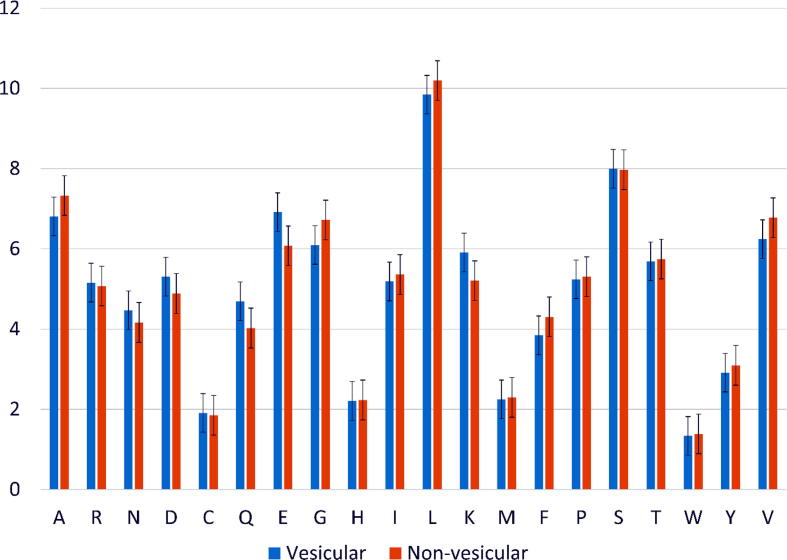
Amino acid composition in vesicular transport and non-vesicular transport proteins.

We calculated the frequency between vesicular transport and non-vesicular transport proteins to analyze the differences between them. Fig. 2 shows the amino acid composition of the vesicular and non-vesicular transport proteins. The error bars on the chart show whether there is a significant difference in the contributions of these amino acids. As shown in this figure, there are not many differences between the amino acid frequencies surrounding these two datasets since they both come from the membrane transport proteins and have a similar structure. Because of this reason, we cannot apply the basic feature sets, e.g. amino acid composition, dipeptide composition in this study. However, some minor differences between two sets of data could be shown, such as amino acids E, K, and Q may play an important role in deciding vesicular transport proteins. On the other hand, amino acid G has a higher frequency and would play a more important role in non-vesicular transport proteins.

**Fig. 3:**
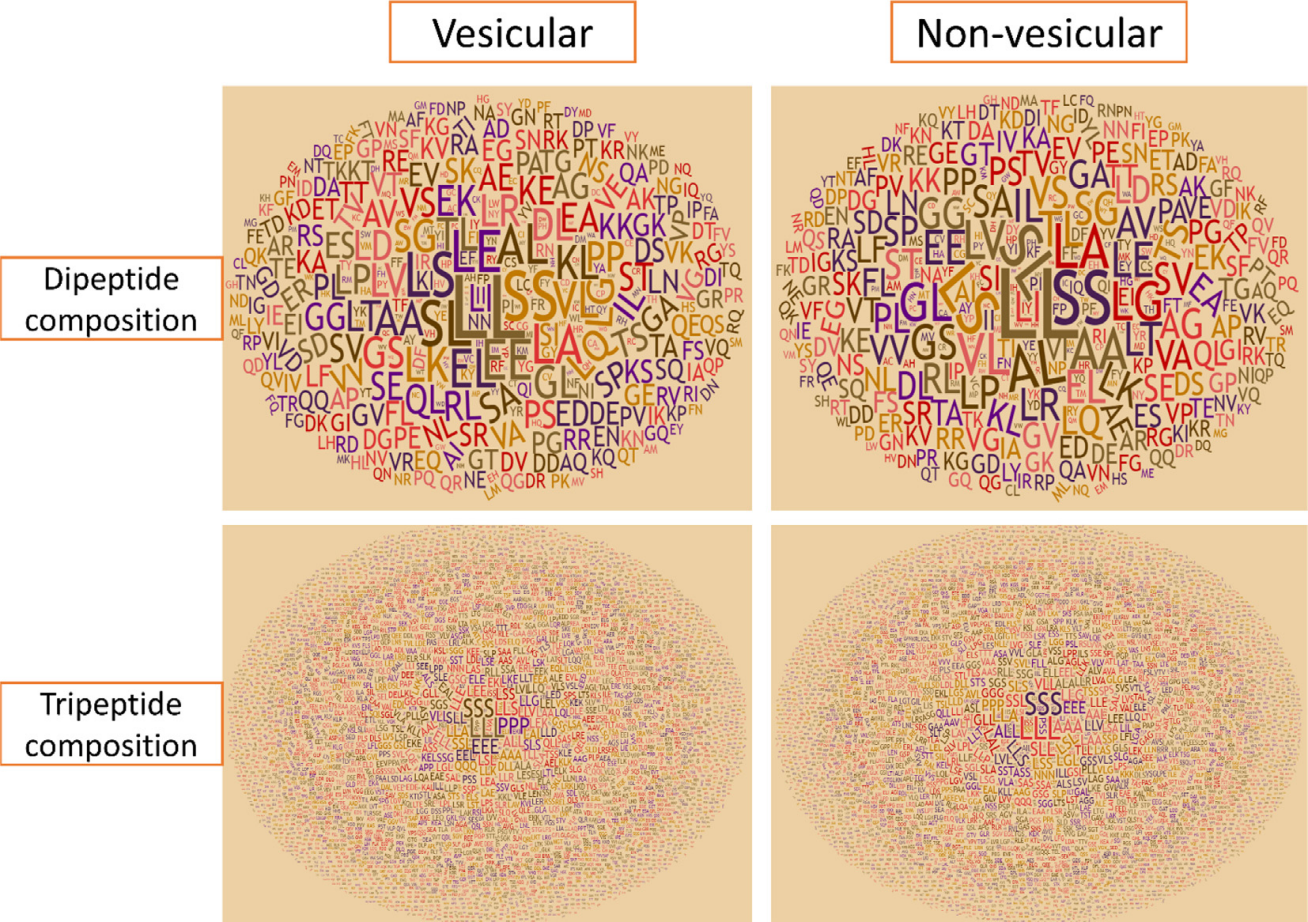
Comparison between vesicular and non-vesicular transport proteins using their dipeptide and tripeptide composition.

We tried to look at the motifs that often appear in protein sequences in the following analysis. Fig. 3 shows the most frequent motifs in vesicular and non-vesicular transport of dipeptide and tripeptide residues. The results show that the protein sequence contained more important motif residues while only containing one residue amino acid composition. Note that this analysis was performed using our training dataset. Dipeptide composition showed that the pairs of LE, EE, and EL are dominant in vesicular transport proteins but less frequent in non-vesicular transport proteins. Regarding tripeptide composition, motifs PPP and EEE are more abundant in the vesicular transport sequences under study. Thus, we are able to discover some motifs to discriminate vesicular transport proteins from general proteins and our model aims to discriminate them according to the sequence information.

### 3.2 Model optimization

Hyper-parameters (e.g., convolutional feature size, fully connected size, kernel size, and so on) optimization has been examined to identify the optimal setup of our model. Firstly, GRU hidden sizes were ranged from 50 to 500 (with step size of 50) to search for the optimal one. After this step, we realized that our model came to the highest performance when GRU sizes came to 250 (training accuracy of 81.6% and MCC of 0.41). The GRU sizes play a role like feature selection technique, which means that we have selected the 250 features in our GRU architecture.

We also investigated the performance results of different fully connected layer sizes. As shown in Table 3, using a bigger size of fully connected layers did not increase the performance, rather it achieved worse results. From this table, the fully connected layer size of 32 performed better than the others. Thereafter, this parameter was used subsequently for the rest of the experiments. This indicated that the big filter size did not have a significant impact on this problem, hence, the simplest filter sizes are required to achieve significant results.

**Table 3:** Performance results of identifying vesicular transport proteins with different fully-connected (FC) layer sizes.

FC sizes	Sensitivity	Precision	Specificity	Accuracy	MCC	AUC
16	39.6	63.4	93.5	81.5	0.40	0.765
32	**40.9**	63.4	93.3	**81.6**	**0.41**	**0.771**
64	34.6	**65.2**	**94.7**	81.4	0.38	0.757
128	40.8	63	93.1	81.5	0.40	0.75
256	38.8	63.2	93.5	81.4	0.39	0.762
512	38.2	63.7	93.8	81.4	0.39	0.76
1024	37.1	64.7	94.2	81.5	0.39	0.757

The bold values are the highest ones in each specific metric.

To evaluate the model’s performance, an independent dataset was used for another testing. To increase the persuasiveness of the problem, we chose the independent dataset from newly discovered proteins (proteins discovered after 2009). This means that we used the old proteins to build a model and evaluate them with new proteins. None of the samples in the independent dataset is contained in the cross-validation dataset and it also has sequences with identity of less than 30%. After performing experiments, our independent test results reached an accuracy of 85.8% and MCC of 0.44. The results between the cross-validation and independent datasets are consistent with less differences. It claims that the optimal hyper-parameters could be used to evaluate the independent dataset and there was not overfitting in our model. In addition, the overfitting was also resolved due to of our dropout layers which had been inserted in the GRU network.

### 3.3 Imbalanced problem solving

A common problem in supervised learning is the imbalance of the dataset, due to the number of negative samples being much higher than the number of positive samples. In the current study, the number of negative samples (non-vesicular transport proteins) is 3.42 times higher than those of positive examples (vesicular transport proteins). A predictor trained by such a highly skewed dataset may introduce inaccuracies in prediction of the vesicular transport as non-vesicular transport ones. Therefore, in our results, the low sensitivity of the methods is due to the larger number of negative examples compared to positive examples.

Recently, there are many techniques to deal with an imbalanced dataset, such as oversampling [44], under-sampling [45], and class weight tuning [46]. Each technique might be suitable for a specific problem and many researchers attempted to evaluate and find the optimal one for their problem. In this study, we also applied those techniques to consider the suitable one for our model. A data pre-processing approach was applied by randomly oversampling the minority class or under-sampling the majority class in the training dataset. By choosing oversampling, we not only have sufficient data for the deep learning method but could also avoid losing valuable information. One concern when using oversampling is that our model will become overfitted in some cases. On the other hand, under-sampling will allow us to attain clean data with no similarity. However, we will lose information through the removal of some of the negative samples. The last method we applied in this study is class weight tuning, in which we kept the original dataset and used weight tuning in the loss function. It is also a good solution and has been used in many deep learning applications. An important note here is that we only applied sampling techniques in the training set but not in the testing set. This ensures the accuracy in using those techniques, making the results more reliable. Table 4 shows the performance results when we applied three imbalanced techniques. We see that the class weight tuning method is superior to the other two. Now we can increase the performance of our model, especially in sensitivity and MCC, which reached 79.2% and 0.52, respectively. It means that we can predict accurately more vesicular transport proteins and increase the quality of the model.

**Table 4:** Comparative performance results among different imbalanced techniques.

Techniques	Sensitivity	Precision	Specificity	Accuracy	MCC	AUC
Oversampling	77.3	47.4	82.5	81.6	0.50	0.849
Undersampling	60.4	46.5	85.8	81.5	0.42	0.781
Class weight tuning	79.2	48.7	82.9	82.3	0.52	0.861

### 3.4 Effectiveness on the other datasets

In this section, we aim to carry out a set of additional experiments to see whether our method works well on different datasets or using different separation way. In the first try, we would like to see whether picking only membrane non vesicular transporters has an impact or general protein. Therefore, we randomly collected a set of general proteins to make it a negative dataset. Note that we excluded all of the vesicular transport proteins and removed all the sequences with identify level of 30%. A set of 12,746 proteins was retrieved and we randomly divided it into cross-validation set and independent dataset with ratio 5:1 (10,898 sequences for cross-validation and 1847 sequences for independent test). Thereafter, a binary classification between vesicular transport protein and general protein has been made using our best GRU architecture. As a result, this model reached an average 5-fold cross-validation sensitivity, precision, specificity, accuracy, and MCC of 58.2%, 41.8%, 83.8%, 79.5%, and 0.37, respectively. Compared to our membrane set’s results (Table 3), it has been consistent. It can be claimed that we can use membrane proteins to represent general proteins with a same-level performance.

Moreover, because we used the newly discovered sequences for independent dataset, we also examined our performance results on a different independent dataset. Contrasting with this independent dataset, we used the “old” protein as new independent dataset and the other proteins as our training set. In total, there were 420 vesicular transport proteins used in this new set. After performing the optimal GRU architecture (Table 2’s parameters), we reached a sensitivity of 67.1%, precision of 51.5%, specificity of 82.4%, accuracy of 79.1%, and MCC of 0.45. It is easy to observe that the performance results were consistent with the selected independent dataset. Therefore, we could claim that our model was efficient in identifying vesicular transport proteins, even with different separation of data.

### 3.5 Comparison with the previous techniques and methods

**Fig. 4:**
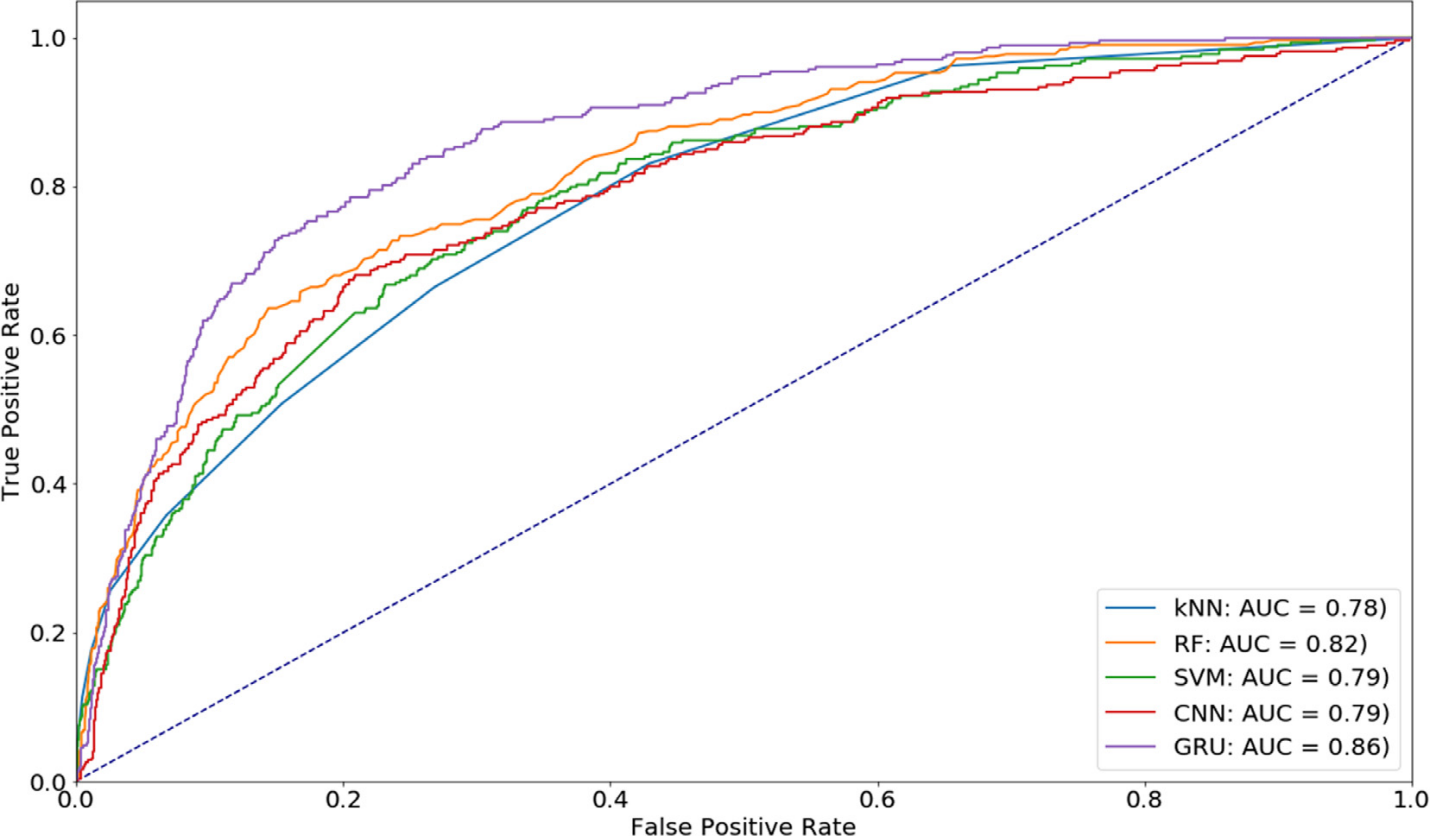
ROC Curves among different methods for identifying vesicular transport proteins.

As shown in Table 2, we have already selected our best layers and parameters for our neural network. In this section, we aim to compare our performance with the previous techniques as well as networks. One of the most efficient methods in this field is to transform PSSM profile from 20*n dimension to 20*20 dimension and feed into neural network. This method has been successfully applied in numerous sequence-based protein function prediction with valuable results such as transport proteins or cytoskeleton motor proteins [35], [47]. A big limitation of this method was that there was no order of information, which our method managed to address. We conducted the comparative performance with three common classifiers: *k*-nearest neighbors (kNN) [48], Random Forest [49], and SVM kernel [50] due to their significant improvements in a lot of similar studies. The next classifier that we would like to compare against is 2D CNN, which is recently considered as one of the best methods to resolve this type of problem [9], [47]. The traditional machine learning algorithms have been implemented by using Python language and Scikit-learn package, while 2D CNN have been implemented by using Keras deep learning library. To have a fair comparison, we tuned the optimal parameters for all these classifiers via a systematic grid-search on the training dataset. To detail, the number of nearest neighbors were ranged from one to ten in kNN (step size = 1), number of trees were ranged from 100 to 500 in Random Forest (step size = 100), and cost was ranged from −5 to 15 (step size = 2), gamma was ranged from 3 to −15 (step size = −2) in SVM to perform a grid search and find the optimal cost and gamma. For 2D CNN, we performed a hyperparameter optimization process to select the optimal number of layers, filters, dropout level, and optimizers. After tuning, we specified the optimal parameters of each classifier as follows: k = 10 in kNN, n_estimators = 100 in Random Forest, cost = 2 and gamma = 0.5 in SVM, number of filters = 128, dropout = 0.1 in 2D CNN. We also used weight tuning in these classifiers to fairly compare with GRU architecture. In summary, the comparative performance among different classifiers was shown in Fig. 4. To see the performance at different levels of threshold, we showed the ROC Curve and AUC in this figure. We readily noted that the performance results of our GRU was also greater than the other methods in most of points. However, the issue posed here is how to maintain GRU's better output when it undergoes many cross-validation tests compared to other methods. To answer this question, we carried out a paired t-test to determine whether other techniques are considerably better or worse, or whether there is even no statistical distinction relative to GRU. The null hypothesis assumes that the real mean difference between the combined metrics is zero and p-value = 0.05 (95% confidence level) determines the statistical significance. After performing statistical test, the p-values were 0.00029, 8.81e-05, 0.00216, and 0.000137 when comparing GRU with kNN, Random Forest, SVM, and 2D CNN, respectively. The low p-values showed that our GRU outperformed the other methods with high confidence level. It can be claimed that the order information of PSSM plays an important role in identifying the protein function in general and vesicular transport in particular. Also, this fill a gap that the previous works could not address even using GRU architecture [26], [36].

Moreover, we also compared our performance results with three different methods: (1) using traditional PSSM features + GRU (to show that the claimed improvement is not merely because of GRUs), (2) using BLSTM which is decidedly more prevalent in the published works for protein applications, and (3) using BLAST [32] which is a general purpose protein function prediction tool as shown in paper [51]. Table 5 shows the comparative performance among different methods. Again, our GRU architecture still outperformed the other ones at the same level of comparison, especially in term of sensitivity, MCC, and AUC.

**Table 5:** Comparative performance results among different protein function prediction methods.

Techniques	Sensitivity	Precision	Specificity	Accuracy	MCC	AUC
Traditional GRU*	70.8	44	81	79.2	0.44	0.848
BLSTM	54.2	55.8	90.9	84.6	0.46	0.846
BLAST	54.1	52.8	89.8	83.6	0.43	0.82
New GRU**	79.2	48.7	82.9	82.3	0.52	0.861

(* traditional PSSM profiles + GRU, ** our GRU architecture).

### 3.6 Releasing benchmark datasets and source codes for re-producing the model

We provided all datasets and source codes on https://github.com/khanhlee/vesicular-gru to make our article simple to replicate. Python language has been used to implement all the deep learning architectures through the Pytorch library [39]. In order to re-implement the technique, readers and scientists can freely access the information and predict their own sequences without a web server. We supplied the highest model in the design phase based on 5-fold cross-validation results. Researchers with restricted programming and machine learning understanding can readily use these resources to accomplish their job.

Furthermore, as shown in a series of latest papers on the growth of prediction techniques, user-friendly and publicly available web servers would improve their effect considerably, leading to an unprecedented revolution in medicinal chemistry [7], [27], [41]. We want our future study to be able to provide a web server for the forecast technique described in this document.

## 4 Conclusion

In this research, we approached an innovative technique for discriminating vesicular transport proteins using GRU and PSSM profiles. With this technique, all the PSSM data can be preserved in deep neural networks to avoid missing data as much as possible. We used 5-fold cross-validation and independent test data (including 319 vesicular transport proteins and 1513 non-vesicular transport proteins) to evaluate performance. Our method showed a 5-fold cross-validation accuracy of 82.3% and MCC of 0.52 for predicting vesicular transport proteins. The accuracy and MCC with independent datasets are 85.8% and 0.44, respectively. This strategy accomplished an obvious enhancement in all assessment metrics compared to the results of the other state-of-the-art techniques. We approached a strong model throughout this research to discover new proteins that highly belong to vesicular transportation proteins. The results of this study could provide a foundation for further studies that could use the GRU and PSSM profiles in computational biology. In addition, scientists can also use our architecture in the future to solve several protein function prediction issues.

## Declaration of Competing Interest

The authors declare that they have no known competing financial interests or personal relationships that could have appeared to influence the work reported in this paper.

## References

[1] Chou K.-C. (2009). Pseudo amino acid composition and its applications in bioinformatics, proteomics and system biology. Curr Proteomics.

[2] Cui X. (2019). UbiSitePred: a novel method for improving the accuracy of ubiquitination sites prediction by using LASSO to select the optimal Chou's pseudo components. Chemom Intell Lab Syst.

[3] Jones D.T. (1999). Protein secondary structure prediction based on position-specific scoring matrices. J Mol Biol.

[4] Le N.Q.K., Ou Y.Y. (2016). Prediction of FAD binding sites in electron transport proteins according to efficient radial basis function networks and significant amino acid pairs. BMC Bioinf.

[5] Kawashima S., Kanehisa M. (2000). AAindex: amino acid index database. Nucleic Acids Res.

[6] Wei L., Chen H., Su R. (2018). M6APred-EL: a sequence-based predictor for identifying N6-methyladenosine sites using ensemble learning. Mol Ther Nucleic Acids.

[7] Le N.Q.K., Ho Q.T., Ou Y.Y. (2017). Incorporating deep learning with convolutional neural networks and position specific scoring matrices for identifying electron transport proteins. J Comput Chem.

[8] Wei L. (2018). Prediction of human protein subcellular localization using deep learning. J Parallel Distrib Comput.

[9] Le N.Q.K., Ho Q.-T., Ou Y.-Y. (2018). Classifying the molecular functions of Rab GTPases in membrane trafficking using deep convolutional neural networks. Anal Biochem.

[10] Liu Y., Edwards R.H. (1997). The role of vesicular transport proteins in synaptic transmission and neural degeneration. Annu Rev Neurosci.

[11] Gissen P., Maher E.R. (2007). Cargos and genes: insights into vesicular transport from inherited human disease. J Med Genet.

[12] Cláudio N., Pereira F.J., Barral D.C. (2001). *Membrane traffic and disease*. eLS.

[13] Suzuki T. (2006). Trafficking of Alzheimer's disease-related membrane proteins and its participation in disease pathogenesis. J Biochem.

[14] Weber M.S., Hohlfeld R., Zamvil S.S. (2007). Mechanism of action of glatiramer acetate in treatment of multiple sclerosis. Neurotherapeutics.

[15] Gannon J., Bergeron J.J.M., Nilsson T. (2011). Golgi and related vesicle proteomics: simplify to identify. Cold Spring Harbor Perspect Biol.

[16] Barile M. (2005). Large scale protein identification in intracellular aquaporin-2 vesicles from renal inner medullary collecting duct. Mol Cell Proteomics.

[17] Hager H.A. (2010). Identification of a novel Bves function: regulation of vesicular transport. EMBO J.

[18] Orci L. (1989). Dissection of a single round of vesicular transport: sequential intermediates for intercisternal movement in the Golgi stack. Cell.

[19] Rohan S. (2006). Gene expression profiling separates chromophobe renal cell carcinoma from oncocytoma and identifies vesicular transport and cell junction proteins as differentially expressed genes. Clin Cancer Res.

[20] Saier M.H., Tran C.V., Barabote R.D. (2006). TCDB the transporter classification database for membrane transport protein analyses and information. Nucleic Acids Res.

[21] Andersson M.X., Sandelius A.S. (2004). A chloroplast-localized vesicular transport system: a bio-informatics approach. BMC Genomics.

[22] Lindquist E., Alezzawi M., Aronsson H. (2014). Bioinformatic indications that COPI- and clathrin-based transport systems are not present in chloroplasts: an arabidopsis model. PLoS ONE.

[23] Kloepper T.H. (2007). An elaborate classification of SNARE proteins sheds light on the conservation of the eukaryotic endomembrane system. Mol Biol Cell.

[24] Kloepper T.H., Kienle C.N., Fasshauer D. (2008). SNAREing the basis of multicellularity: consequences of protein family expansion during evolution. Mol Biol Evol.

[25] Le N.Q.K., Nguyen V.-N. (2019). SNARE-CNN: a 2D convolutional neural network architecture to identify SNARE proteins from high-throughput sequencing data. PeerJ Comput Sci.

[26] Pfeiffenberger E., Bates P.A. (2018). Predicting improved protein conformations with a temporal deep recurrent neural network. PLoS ONE.

[27] Le N.Q.K., Yapp E.K.Y., Yeh H.-Y. (2019). ET-GRU: using multi-layer gated recurrent units to identify electron transport proteins. BMC Bioinf.

[28] Le N.Q.K. (2019). Fertility-GRU: identifying fertility-related proteins by incorporating deep-gated recurrent units and original position-specific scoring matrix profiles. J Proteome Res.

[29] Hill S.T. (2018). A deep recurrent neural network discovers complex biological rules to decipher RNA protein-coding potential. Nucleic Acids Res.

[30] Consortium U. (2014). UniProt: a hub for protein information. Nucleic Acids Res.

[31] Ashburner M. (2000). Gene ontology: tool for the unification of biology. Nat Genet.

[32] Altschul S.F. (1997). Gapped BLAST and PSI-BLAST: a new generation of protein database search programs. Nucleic Acids Res.

[33] Mapes N.J. (2019). Residue adjacency matrix based feature engineering for predicting cysteine reactivity in proteins. Comput Struct Biotechnol J.

[34] Kroncke B.M. (2019). Protein structure aids predicting functional perturbation of missense variants in SCN5A and KCNQ1. Comput Struct Biotechnol J.

[35] Le N.Q.K., Sandag G.A., Ou Y.-Y. (2018). Incorporating post translational modification information for enhancing the predictive performance of membrane transport proteins. Comput Biol Chem.

[36] Li Z., Yu Y. (2016). Protein secondary structure prediction using cascaded convolutional and recurrent neural networks. Proceedings of the Twenty-Fifth International Joint Conference on Artificial Intelligence.

[37] Chen L. (2019). Classification of widely and rarely expressed genes with recurrent neural network. Comput Struct Biotechnol J.

[38] Lyu C. (2017). Long short-term memory RNN for biomedical named entity recognition. BMC Bioinf.

[39] Paszke, A., et al., Automatic differentiation in PyTorch. 2017.

[40] Srivastava N. (2014). Dropout: a simple way to prevent neural networks from overfitting. J Mach Learn Res.

[41] Le N.Q.K. (2019). iEnhancer-5Step: identifying enhancers using hidden information of DNA sequences via Chou's 5-step rule and word embedding. Anal Biochem.

[42] Friedman J., Hastie T., Tibshirani R. (2001). The elements of statistical learning. Springer series in statistics.

[43] Chou K.C. (2001). Prediction of protein cellular attributes using pseudo-amino acid composition. Proteins Struct Funct Bioinf.

[44] Chawla N.V. (2002). SMOTE: synthetic minority over-sampling technique. J Artif Intell Res.

[45] Liu X.-Y., Wu J., Zhou Z.-H. (2009). Exploratory undersampling for class-imbalance learning. IEEE Trans Syst Man Cybern Part B (Cybernetics).

[46] Dong Q., Gong S., Zhu X. (2018). Imbalanced deep learning by minority class incremental rectification. IEEE Trans Pattern Anal Mach Intell.

[47] Le N.Q.K. (2019). iMotor-CNN: identifying molecular functions of cytoskeleton motor proteins using 2D convolutional neural network via Chou's 5-step rule. Anal Biochem.

[48] Keller J.M., Gray M.R., Givens J.A. (1985). A fuzzy k-nearest neighbor algorithm. IEEE Trans Syst Man Cybern.

[49] Liaw A., Wiener M. (2002). Classification and regression by randomForest. R News.

[50] Hearst M.A. (1998). Support vector machines. IEEE Intell Syst Their Appl.

[51] Jiang Y. (2016). An expanded evaluation of protein function prediction methods shows an improvement in accuracy. Genome Biol.

